# Estimation of Ground PM_2.5_ Concentrations using a DEM-assisted Information Diffusion Algorithm: A Case Study in China

**DOI:** 10.1038/s41598-017-14197-z

**Published:** 2017-11-14

**Authors:** Lei Ma, Yu Gao, Tengyu Fu, Liang Cheng, Zhenjie Chen, Manchun Li

**Affiliations:** 1 Jiangsu Provincial Key Laboratory of Geographic Information Science and Technology, Nanjing University, Nanjing, 210023 China; 2 School of Geographic and oceanographic sciences, Nanjing University, Nanjing, 210023 China; 3 Collaborative Innovation Center for the South Sea Studies, Nanjing University, Nanjing, 210023 China

## Abstract

When estimating national PM_2.5_ concentrations, the results of traditional interpolation algorithms are unreliable due to a lack of monitoring sites and heterogeneous spatial distributions. PM_2.5_ spatial distribution is strongly correlated to elevation, and the information diffusion algorithm has been shown to be highly reliable when dealing with sparse data interpolation issues. Therefore, to overcome the disadvantages of traditional algorithms, we proposed a method combining elevation data with the information diffusion algorithm. Firstly, a digital elevation model (DEM) was used to segment the study area into multiple scales. Then, the information diffusion algorithm was applied in each region to estimate the ground PM_2.5_ concentration, which was compared with estimation results using the Ordinary Kriging and Inverse Distance Weighted algorithms. The results showed that: (1) reliable estimate at local area was obtained using the DEM-assisted information diffusion algorithm; (2) the information diffusion algorithm was more applicable for estimating daily average PM_2.5_ concentrations due to the advantage in noise data; (3) the information diffusion algorithm required less supplementary data and was suitable for simulating the diffusion of air pollutants. We still expect a new comprehensive model integrating more factors would be developed in the future to optimize the interpretation accuracy of short time observation data.

## Introduction

Aerosols are solid particles or liquid droplets suspended in the atmosphere^1^. Studies suggest that long-time exposure to environments with high PM_2.5_ (particulate matter with an aerodynamic diameter of <2.5 μm) concentrations leads to a higher prevalence of cardiovascular and respiratory diseases^2,3^, with the highest incidence rate in developing countries. The acceleration of urbanization in China has led to increasingly severe environmental issues caused by excessive emission of inhalable particles^4–6^. In recent years, an increasing number of studies have been performed to estimate ground PM_2.5_ concentrations based on remote-sensing aerosol optical thickness (AOD)^7–12^. Such models usually require abundant and accurate supplementary data (such as weather, terrain, and land cover data) to improve estimation accuracy. However, this data is often difficult to obtain, and variations usually exist in spatial and temporal resolutions, thus affecting the accuracy of the models. In addition, most models are region specific^13,14^, which restricts their applications.

The spatial interpolation algorithm can be used to overcome the issues relating to data acquisition and model development when estimating ground PM_2.5_ concentrations. In recent years, the air quality monitoring network has been gradually improved^4^, leading to a continued increase in estimates of PM_2.5_ concentration and risk assessments based on site monitoring data^15,16^. To our knowledge, several studies have estimated PM_2.5_ concentrations on different scales using interpolation algorithms^17–19^. However, there is still a lack of PM_2.5_ monitoring sites, and their spatial distribution is heterogeneous^20^, making it hard to obtain reliable results using traditional interpolation algorithms (such as Inverse Distance Weighted and Ordinary Kriging). Furthermore, PM_2.5_ spatial distribution is strongly correlated to various factors such as the terrain, vegetation, population density, and economic development level^1,21,22^, yet traditional interpolation algorithms are relatively fixed, and typically only consider one factor for calculation^23^. Therefore, it is difficult to combine the factors affecting PM_2.5_ spatial distribution with the algorithms. These shortcomings restrict the accuracy of traditional interpolation algorithms in ground PM_2.5_ concentration estimation.

In recent years, the information diffusion algorithm, a normal diffusion interpolation model based on the fuzzy mapping philosophy, has shown a certain advantage in its application to small-sample and nonlinear interpolation^24,25^. The information diffusion algorithm transforms the original information to value samples of the fuzzy set, and then assigns information of single-value samples to different discrete points based on the diffusion functions^26,27^. In this way, reliable results can be produced even if the underlying physical processes are not fully understood^28^. This algorithm has been widely applied to various fields such as risk assessment^29,30^ and sea depth estimation^31^. However, the heterogeneous spatial distribution of monitoring data still affects the estimation accuracy of the information diffusion algorithm. Wang *et al*.^32^ improved this by calculating the optimal window width in inhomogeneous samples (that is, using the monitoring data of areas adjacent to points for estimation, instead of all monitoring data, for interpolation calculation). The work of Dong *et al*.^33^ indicated that PM_2.5_ spatial distribution is correlated with elevation, and that collinearity exists between elevation and multiple factors characterized by human activities^1^. Hence, this study used the information diffusion algorithm to estimate ground PM_2.5_ concentrations by assuming the segmentation results of a digital elevation model (DEM) as the interpolation window widths. We exploited the advantages of the information diffusion algorithm to combine elevations affecting PM_2.5_ spatial distribution with the algorithm. As a result, the problem of a lack of data samples was removed, supplementary data acquisition was simplified, and model improvement was simpler than when using traditional algorithms.

This study estimated ground PM_2.5_ concentrations in a study area in China based on both ground-measured PM_2.5_ data and DEM data using the information diffusion algorithm to address the data incompleteness issue. The purpose of this study was to overcome the shortcomings of traditional interpolation algorithms, and provide a reliable method for rapidly monitoring ground PM_2.5_ concentrations on a large scale. The main goals were to: (1) verify the effectiveness of the DEM-assisted information diffusion algorithm for estimating ground PM_2.5_ concentrations; (2) compare estimation accuracy between the DEM-assisted information diffusion algorithm, the Inverse Distance Weighted method, and the Ordinary Kriging method to verify the reliability of the information diffusion algorithm; and (3) analyze the applicability of the DEM-assisted information diffusion algorithm for monitoring data on different time scales.

## Study area and data

### Study area

**Figure 1:**
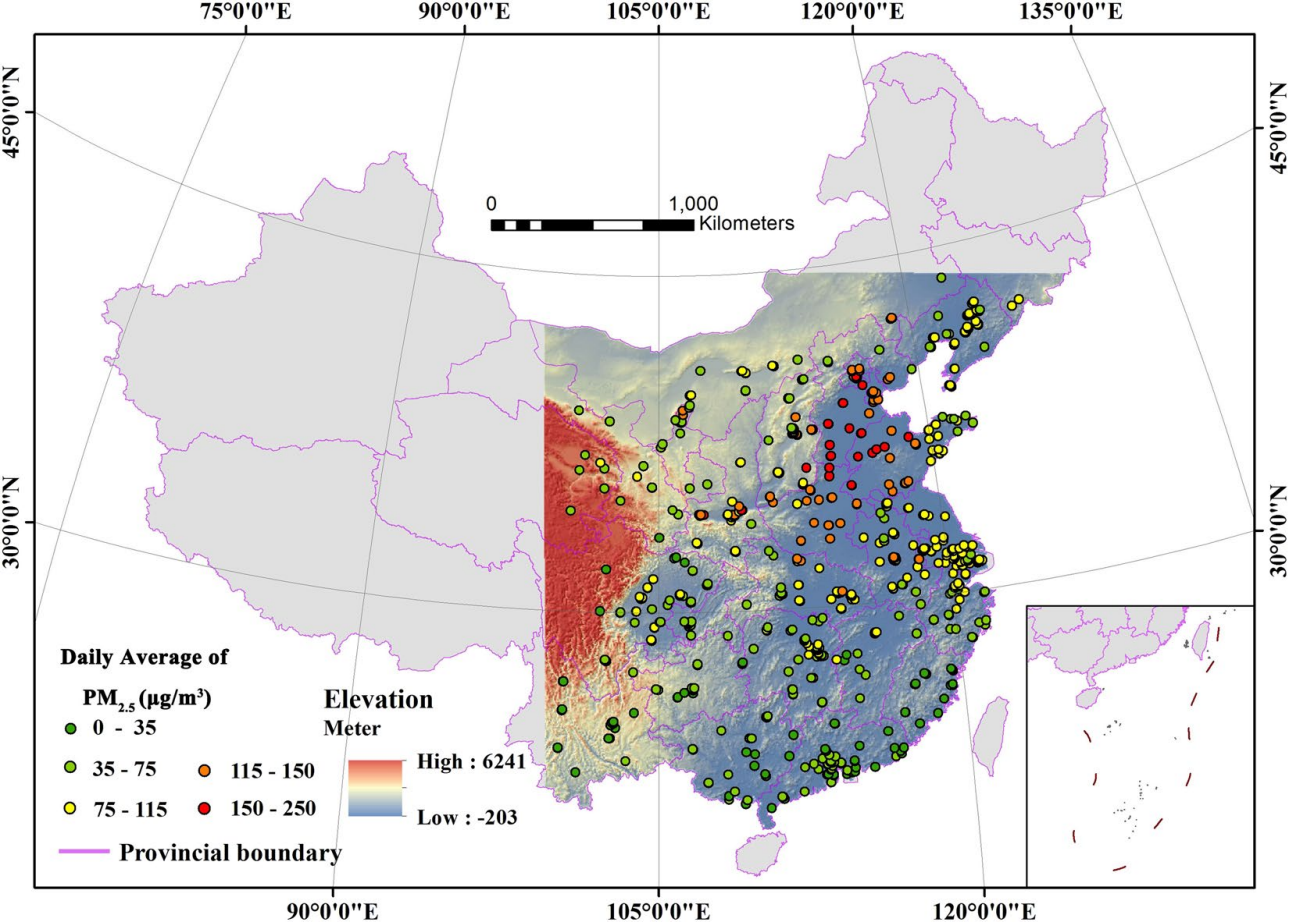
Elevation and spatial distribution of PM_2.5_ monitoring sites in the study area. (Created by ArcMap, version 10.2, http://www.esri.com/).

The study area lies between 19.98° N and 44.78° N, and 99.53° E and 129.33° E in mainland China. It covers a land area of 4,870,000 km^2^, 51% of China’s total area (Fig. 1). The study area contains the eastern coastal area, which boasts the most rapidly developing economy, the highest levels of industrialization and urbanization, and the densest cluster of urban agglomerations in the country. In the past few years, the air quality has severely deteriorated in all provinces and municipalities of the study area, greatly affecting the lives of residents^34^. This phenomenon is caused by anthropogenic emission sources from urban expansion, industrial emissions, and excessive use of vehicles^3,35^. Therefore, this area is an important site for studying ground PM_2.5_ concentrations, aided by the gradual implementation of a ground PM_2.5_ concentration monitoring network since 2013. According to segmentation result, the study site was divided into seven geographical subareas with different range of elevation (Table 1). High altitude areas with plateau and mountain are mainly concentrated in three regions, followed by Tibetan Plateau area (average altitude 3300 m), Yunnan-Guizhou Plateau area (average altitude 1712m), Inner Mongolia-Loess Plateau area (average altitude 1279 m). There are some hills in Northeastern area (average altitude 499 m), Southeastern coastal area (average altitude 311 m) and Central area (average altitude 743 m), while the plain mainly distributed in Eastern coastal area (average altitude 63 m). Therefore, you may find the study area has a large altitude range and diverse landscape types, making it suitable for analyzing the relationship between ground PM_2.5_ concentration and elevation.

**Table 1:** Seven geographical subareas of the study area divided by segmenting the elevation.

No.	Geographical Subarea	Provinces and Municipalities	Altitude (m)	
			Average	Range
1	Northeastern area	Liaoning province, south Jilin province, and north Hebei province	499	−203–2322
2	Eastern coastal area	Beijing city, Shanghai city, Tianjin city, Hebei province, Shandong province, Anhui province, and east Henan province	63	−4–1289
3	Southeastern coastal area	Zhejiang province, Fujian province, Jiangxi province, Hunan province, Guangdong province, Guangxi Zhuang Autonomous Region, and east Hubei province	311	−8–2122
4	Central area	Chongqing city, west Henan province, south Shaanxi province, east Hubei province, and east Sichuan province	743	63–2795
5	Inner Mongolia-Loess Plateau area	Inner Mongolia Autonomous Region, Ningxia Hui Autonomous Region, Shanxi province, and north Shaanxi province	1279	376–3069
6	Yunnan-Guizhou Plateau area	Yunnan province, Guizhou province, and south Sichuan province	1712	132–4638
7	Tibetan Plateau area	Gansu province, northeast Sichuan province, and east Qinghai province	3300	630–6241

### PM2.5 monitoring site data

We used hourly PM_2.5_ monitoring data provided by the China Environment Monitoring Center (http://106.37.208.233:20035). Data was collected from 763 monitoring sites from January to December 2015 (Fig. 1), and PM_2.5_ concentrations were measured using the Tapered Element Oscillating Microbalance (TEOM) method^36^. Based on hourly PM_2.5_ monitoring data, daily, monthly, and quarterly average data was obtained to test the applicability of the information diffusion algorithm on different time scales.

### DEM data

To consider the impact of elevation on PM_2.5_ spatial distribution, we obtained the DEM product generated during the Shuttle Radar Topography Mission (SRTM) from the United States Geological Survey (USGS) (ftp://e0srp01u.ecs.nasa.gov/srtm/version2/SRTM3). This product was used by the National Aeronautics and Space Administration (NASA) to measure PM_2.5_ concentrations in over 80% of the land area between latitudes 56° S and 60° N, with a spatial resolution of 90 m. Here, we used ArcGIS10.2 to bridge single-frame product data and obtain DEM data for the whole study area.

### Methodology

**Figure 2:**
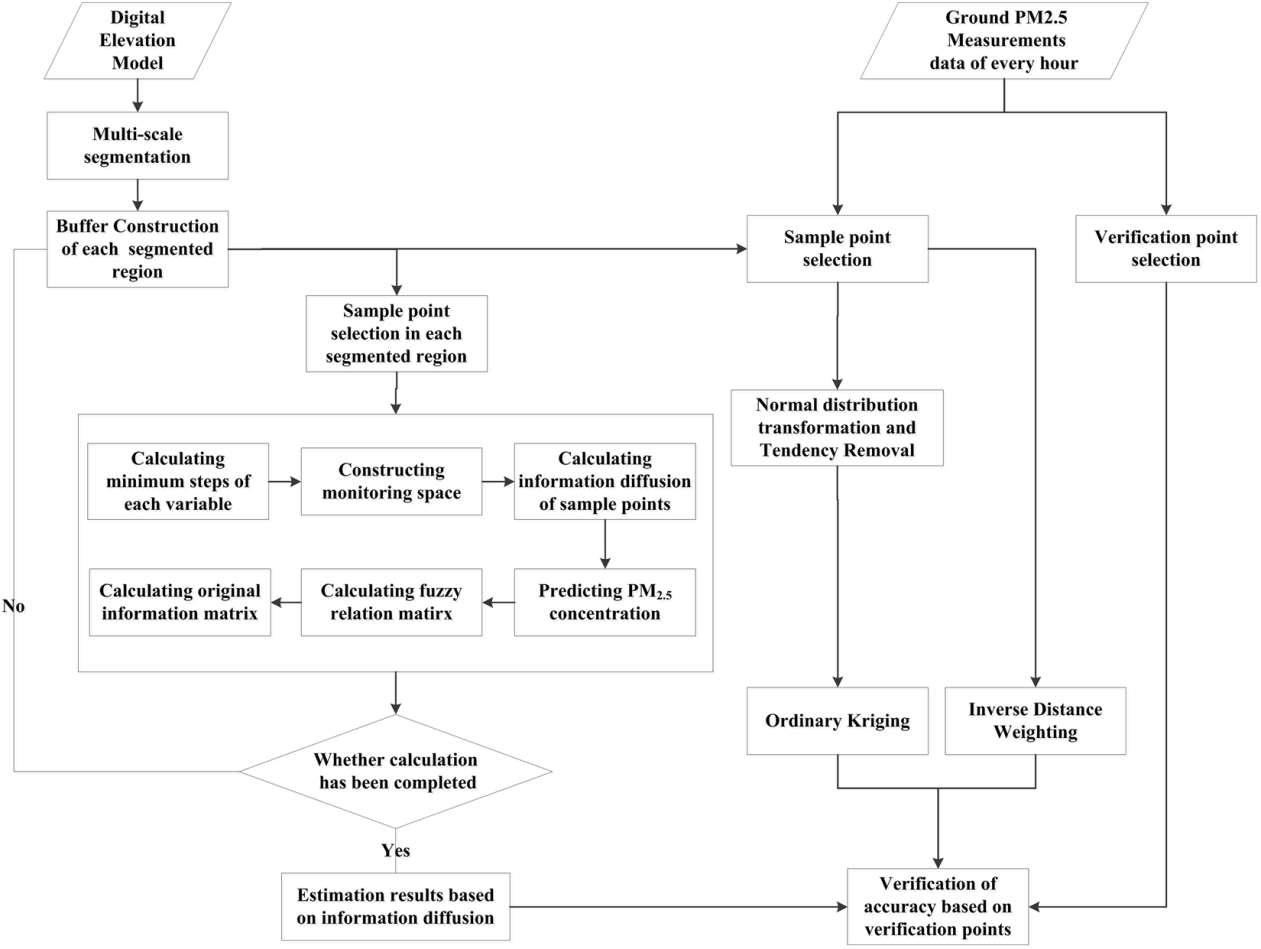
Flowchart of the proposed approach.

The multi-scale segmentation algorithm was combined with the information diffusion algorithm to estimate ground PM_2.5_ concentrations in the study area using ground-measured PM_2.5_ data, based on the assumption that ground PM_2.5_ spatial distribution is highly correlated with elevation. The main methods were as follows: (1) 10-fold cross-validation was used to assess the accuracy of models, and the monitoring data set was randomly split into ten equal-sized data sets, with one group as the validation set and the remaining nine groups together forming the training set; (2) multi-scale segmentation based on the DEM, where the study area was segmented into multiple regions of homogeneous elevation, generating interpolation windows for the information diffusion algorithm; (3) in each segmented region, the information diffusion algorithm was used to estimate ground PM_2.5_ concentrations based on daily, monthly, and quarterly average PM_2.5_ data from ground monitoring sites; (4) the Ordinary Kriging and Inverse Distance Weighted algorithms were used to estimate ground PM_2.5_ concentrations based on the same training set; (5) the experiments were performed ten times, and accuracy assessment indexes of different algorithms were calculated using the validation set in each experiment; and (6) the mean values of the accuracy assessment indexes were calculated, and the differences between the information diffusion algorithm and traditional interpolation algorithms were explained. The flowchart of the full analysis procedure is illustrated in Fig. 2.

### Multi-scale segmentation

A recent study showed that the different spatial scale of characteristic variables could be affecting the PM_2.5_ concentration estimation performance of the interpolation algorithm^37^. Thus, it is necessary to find the optimized spatial scale. Because DEM data was included to consider the impact of elevation on PM_2.5_ spatial distribution, remote-sensing image segmentation was used to determine the optimized spatial scale and ensure the degree of automation of the entire procedure. Multi-scale segmentation is one of the most popular remote-sensing image segmentation algorithms^38,39^. It is a bottom-up region integration process starting from the pixel layer. Image objects are integrated into larger image objects layer by layer, producing segmentation results of different segmentation scales. Based on this approach, this study used the eCognition 9.0 multi-scale segmentation algorithm to perform data segmentation based on the obtained DEM data. Segmentation divided the study area into multiple segments with small changes in internal elevation. During interpolation, the segments were used as windows to select sample points and integrate elevation data with the information diffusion algorithm.

### Information diffusion

Information diffusion is a mathematical model proposed to address the information incompleteness issue during assessment of small-sample events such as natural disasters^27^. Based on this approach, this study regarded PM_2.5_ monitoring data as incomplete sample data, used the diffusion function to binarize the fuzzy sets of PM_2.5_ data, and performed interpolation to obtain homogeneously distributed grid point data. Data of ground PM_2.5_ concentration variability was then obtained for the whole study area. The following elaborates on the information diffusion algorithm procedure.

1. Constructing the monitoring spaces
  To effectively construct the monitoring space, the minimum step is calculated for each variable in the information diffusion model, and the range of monitoring values is discretized to an equal-interval discrete space, namely, the monitoring space.
  1 $$\Delta x=\mathop{\min }\limits_{{x}_{i}\ne {x}_{j}}\{|{x}_{i}-{x}_{j}||i,j=1,2,\ldots ,n\}$$
  2 $$\Delta y=\mathop{\min }\limits_{{y}_{i}\ne {y}_{j}}\{|{y}_{i}-{y}_{j}||i,j=1,2,\ldots ,n\}$$
  3 $$\Delta z=\mathop{\min }\limits_{{z}_{i}\ne {z}_{j}}\{|{z}_{i}-{z}_{j}||i,j=1,2,\ldots ,n\}$$
  where *x*_*i*_, *y*_*i*_, and *z*_*i*_ are coordinates of Sample Point *i*, and *x*_*j*_, *y*_*j*_, and *z*_*j*_ are coordinates of Sample Point *j*. According to the minimum steps given by the above equations, the monitoring space can be constructed as $(U\times V\times W)$, in which $U=\{{u}_{i}|i=1,2,\ldots ,m\}$, $V=\{{v}_{j}|j=1,2,\ldots ,t\}$, and $W=\{{w}_{k}|k=1,2,\ldots ,t\}$. In these variables, ${u}_{i}={u}_{0}+i\Delta x$, ${v}_{j}={v}_{0}+j\Delta y$, and ${w}_{k}={w}_{0}+k\Delta w$. The default values of ${u}_{0}$, ${v}_{0}$, and ${w}_{0}$ are 0.
  We assumed that the minimum steps of the monitoring space in three dimensions are $\Delta u=\Delta x$, $\Delta v=\Delta y$, and $\Delta w=\Delta z$, respectively. The extreme values monitored in each dimension are: ${a}_{x}=\,\min \,\{{x}_{i}\}$, ${b}_{x}=\,\max \,\{{x}_{i}\}$; ${a}_{y}=\,\min \,\{{y}_{i}\}$, ${b}_{y}=\,\max \,\{{y}_{i}\}$; ${a}_{z}=\,\min \,\{{z}_{i}\}$, ${b}_{z}=\,\max \,\{{z}_{i}\}$, respectively. The simple coefficient *h* can be given by the averaging model^24^, as follows:
  4 $$h=\{\begin{array}{c}\begin{array}{c}\begin{array}{cc}0.8146(b-a) & n=5\end{array}\\ \begin{array}{cc}0.5690(b-a) & n=6\end{array}\end{array}\\ \begin{array}{cc}0.4560(b-a) & n=7\end{array}\\ \begin{array}{cc}0.3860(b-a) & n=8\end{array}\\ \begin{array}{c}\begin{array}{cc}0.3362(b-a) & n=9\end{array}\\ \begin{array}{cc}0.2986(b-a) & n=10\end{array}\\ \begin{array}{cc}\frac{2.6851(b-a)}{n-1} & n > 10\end{array}\end{array}\end{array}$$
  where $a={a}_{x},{a}_{y},{a}_{z}$_,_ $b={b}_{x},{b}_{y},{b}_{{\rm{z}}}$; $n$ is the total number of samples; and $h$ is the diffusion factor.
2. Calculating the information diffusion of sample points
  Assuming that the diffusion information of the PM_2.5_ monitoring point $({x}_{l},{y}_{l},{z}_{l})$ in the monitoring space $Q(u,v,w)$ is ${q}_{l}({u}_{i},{v}_{j},{w}_{k})$, it can be given by:
  5 $$\begin{array}{rcl}{q}_{l}({u}_{i},{v}_{j},{w}_{k}) & = & \frac{1}{{h}_{x}\sqrt{2\pi }}\exp \,[-\frac{{({u}_{i}-{x}_{l})}^{2}}{2{h}_{x}^{2}}]\times \frac{1}{{h}_{y}\sqrt{2\pi }}\exp \,[-\frac{{({v}_{j}-{y}_{l})}^{2}}{2{h}_{y}^{2}}]\\  &  & \times \,\frac{1}{{h}_{z}\sqrt{2\pi }}\exp \,[-\frac{{({w}_{k}-{z}_{l})}^{2}}{2{h}_{z}^{2}}]\end{array}$$
  where *h*_*x*_, *h*_*y*_, and *h*_*z*_ can be given by Equation (4); *l* represents the number of sample points; and ${u}_{i},{v}_{j},{w}_{k}$ represents the monitoring space ($i=1,2,\ldots ,m$, $j=1,2,\ldots ,t$, and $k=1,2,\ldots ,s$).
3. Calculating the original information matrix
  The original information matrix $Q$ can be given by calculating the information diffusion of all monitoring points in the monitoring space:
  6 $${Q}_{ijk}=\sum _{l=1}^{n}{q}_{l}({u}_{i},{v}_{j},{w}_{k})$$
  where ${q}_{l}({u}_{i},{v}_{j},{w}_{k})$ can be obtained by Equation (5) and *l* represents the number of sample points. The original information matrix ${\{{Q}_{ijk}\}}_{m\times t\times s}$ can be calculated using Equation (6).
4. Calculating the fuzzy relationship matrix
  A causal fuzzy relationship matrix can be obtained from the original information matrix $Q$. In this study, we transformed the original information matrix ${\{{Q}_{ijk}\}}_{m\times t\times s}$ into the fuzzy matrix ${\{{r}_{ijk}\}}_{m\times t\times s}$ based on the ${R}_{f}$ model^24^.
  7 $$\{\begin{array}{ll}{S}_{k}=\mathop{\max }\limits_{1\le i\le m,1\le j\le t}{Q}_{ijk}, & k=1,2,\ldots ,s\\ {\mu }_{k}({u}_{i},{v}_{j})=\frac{{Q}_{ijk}}{{S}_{k}}, & i=1,2,\ldots ,mandj=1,2,\ldots ,t\\ {r}_{ijk}={\mu }_{k}({u}_{i},{v}_{j}), & i=1,2,\ldots ,mandj=1,2,\ldots ,tandk=1,2,\ldots ,s\end{array}$$
5. Interpolation calculation

The fuzzy set centroid can be calculated to obtain the estimated ground PM_2.5_ concentration:

8 $$R=\sum _{z\in w}(\frac{{r}_{ijk}}{{w}_{k}})$$

where $W=\{{w}_{k}|k=1,2,\ldots s\}$ and ${r}_{ijk}$ can be given by Equation (7), in which $i=1,2,\ldots m$ and $j=1,2,\ldots t$.

To smooth PM_2.5_ concentration variations in the information diffusion algorithm results, we constructed a buffer up to 100 km long (this was verified as the maximum distance over which the interpolation method could generate a better result than remote-sensing based methods^18^) in each segmented region to increase the number of sample points in each region.

### Inverse Distance Weighted algorithm

The Inverse Distance Weighted algorithm is based on the proximity-similarity principle, i.e., the closer two objects are to one another, the more similar their properties, and the further they are from one another, the more different their properties. The algorithm assumes that the impact of unknown points on to-be-estimated points diminishes as the distance increases. The general formula of the interpolation algorithm is as follows:

9 $$\hat{Z}({S}_{0})=\sum _{i=1}^{N}{\lambda }_{i}Z({S}_{i})$$

where $\hat{Z}({S}_{0})$ is the estimated value of the to-be-estimated point ${S}_{0}$; $N$ is the number of sample points around the to-be-estimated points that are required in the estimation process;${\lambda }_{i}$ is the weight of all sample points in the calculation process; and $Z({S}_{i})$ is the sample value of ${S}_{i}$. The weight can be given by:

10 $${\lambda }_{i}={d}_{i0}^{-p}/\sum _{i=1}^{N}{d}_{i0}^{-p},\sum _{i=1}^{N}{\lambda }_{i}=1$$

where $p$ is a parameter. The optimal value can be determined by acquiring the minimum value of the root-mean-square error (RMSE). In addition, the Inverse Distance Weighted algorithm was implemented by the land statistic module of ArcGIS10.2.

### Ordinary Kriging interpolation

The Ordinary Kriging algorithm is the best linear unbiased prediction (BLUP) for to-be-estimated points based on the structured characteristics of sample points^40^. If the structured variable $Z$ is not constant and the mathematical expectation is unknown, the Ordinary Kriging algorithm can be used for interpolation calculation. It is implemented as follows:

11 $${Z}^{\ast }({x}_{0})=\sum _{i=1}^{n}{\omega }_{i}Z({x}_{i})$$

where $Z({x}_{i})$ represents the sample points of interpolated values around the to-be-estimated points; ${Z}^{\ast }({x}_{0})$ is the unbiased prediction (with a constant mathematical expectation) of $Z({x}_{i})$; ${\omega }_{i}$ is the weight of the ${i}^{th}$ sample point, and $\sum _{i=1}^{n}{\omega }_{i}=1$. When the Ordinary Kriging algorithm is used for calculation, data should first be verified by normal distribution verification and transformation, abnormal value verification and removal, and trend verification and removal, in order to improve the estimation accuracy. The Ordinary Kriging was also conducted in the land statistic module of ArcGIS10.2.

### Accuracy assessment

This study used the accuracy verification method based on ground monitoring sites to assess the validity of spatial interpolation. We extracted the estimation results of validation points and then compared monitoring values with estimation results. The main indicators used for accuracy assessment included the absolute error (AE), root mean squared error (RMSE), and range of absolute error (RAE). AE represents the absolute deviation between the estimation result and the monitoring data. RMSE represents the mean deviation between estimated and monitored values, which indicates the reliability of the interpolation model. Because the experiments were performed ten times, the mean values of the accuracy assessment indicators were assessed for the different algorithms. The above three indicators are calculated as follows:

12 $$AE=|O-S|$$

13 $$RAE={E}_{\max }-{E}_{\min }$$

14 $$RMSE=\sqrt{\{\frac{{[{({O}_{1}-{S}_{1})}^{2}+{({O}_{2}-{S}_{2})}^{2}+\cdots ({O}_{n}-{S}_{n})]}^{2}}{n}\}}$$

where *O* represents the monitoring data and *S* represents the estimation data; *E*_max_ and *E*_min_ represent the maximum and minimum AEs, respectively; and *n* represents the total number of samples. Because the number of validation points in each experiment was more than one, the mean value of AE (called MAE below) was calculated for each experiment.

Following the above steps, this study used the information diffusion, Ordinary Kriging, and Inverse Distance Weighted algorithms to estimate ground PM_2.5_ concentrations. Then, a comparative analysis was performed between the estimates and the monitoring results of the validation points. To study the applicability of different algorithms for different time scales, calculations were based on the daily average data of January 2, 2015, monthly average data of December 2015, and average data of the winter (January to February) of 2015.

## Results

### Segmentation of homogeneous-elevation regions

**Figure 3:**
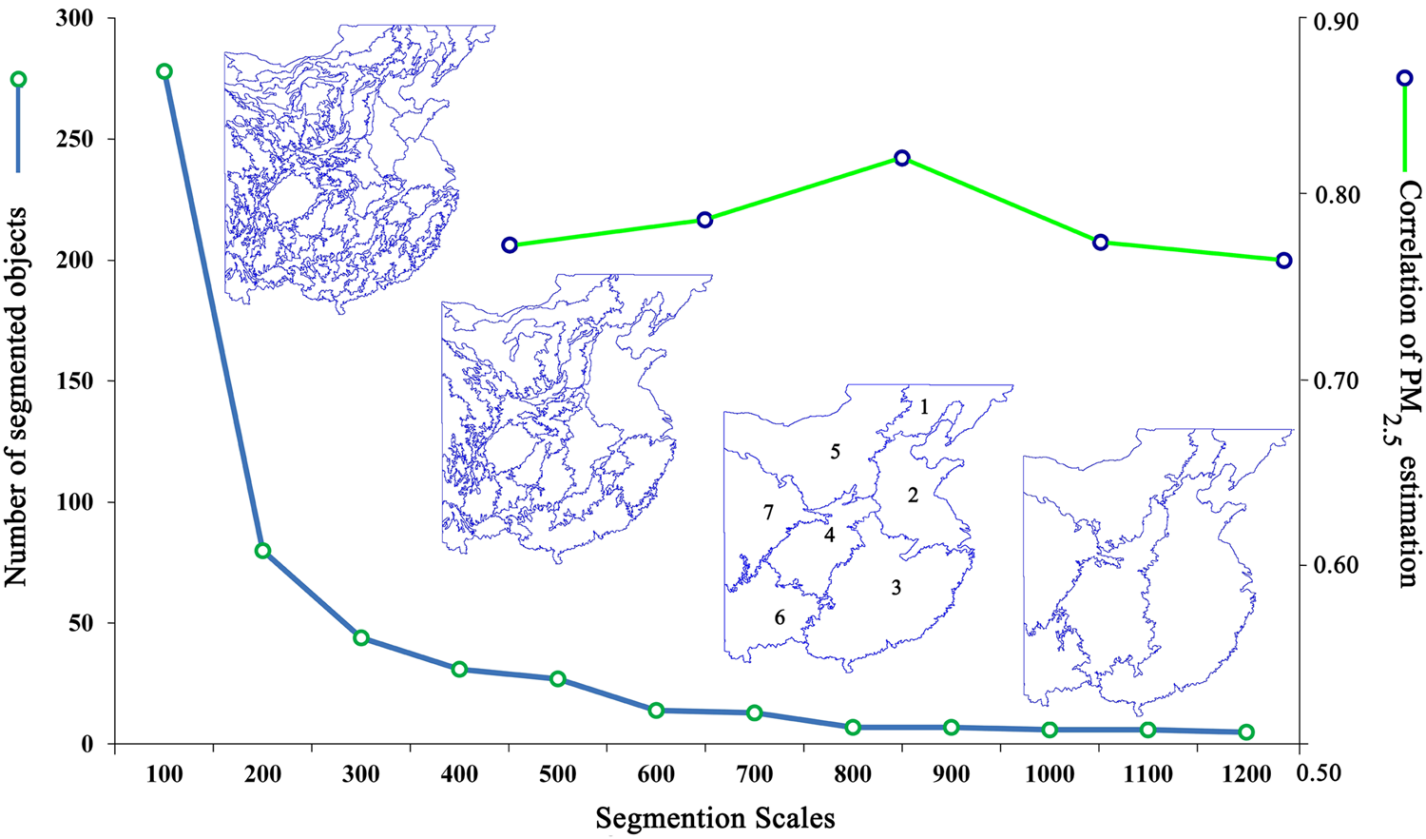
Multi-scale segmentation results of homogeneous-elevation regions, and PM2.5 estimate correlations for different segmentation scales. (Created by ArcMap, version 10.2, http://www.esri.com/).

Using the multi-scale segmentation technique, segmentation scales were continuously optimized to obtain segmentation results at different scales. Figure 3 shows that the number of segments in the study area diminished as the segmentation scale increased. When a small segmentation scale was selected, there were many small segments, and segment homogeneity was high. As the segmentation scale increased, the number of segments decreased, segments with a large variation in elevation characteristics remained approximately the same, and small segments were combined. When the segmentation scale was less than 400 (dimensionless unit), some segments did not have sample points (Fig. 3). When the segmentation scale was between 400 and 800, correlations between the PM_2.5_ concentration estimates increased (from 0.77 to 0.79). When the segmentation scale reached 800, the correlation was 0.83. When the segmentation scale exceeded 800, the correlations of results decreased. To ensure sufficient sample points for calculation in each segment, and to achieve high accuracy, we selected 800 as the optimal segmentation scale based on the above analysis and the calculation requirements. According to Fig. 3, the study area was divided into seven regions of homogeneous elevation (Table 1). The elevations varied slightly in each segmented region and their elevation characteristics were similar, providing appropriate calculation windows for an analysis of ground PM_2.5_ concentration estimates.

### Ground PM_2.5_ concentration estimation

#### Accuracy analysis

Table 2 shows that the correlations of estimates derived from the information diffusion algorithm were more accurate than those of the Ordinary Kriging and Inverse Distance Weighted algorithms based on daily, monthly, and quarterly average data. The information diffusion algorithm showed a slight advantage for global correlation between estimation results (daily average: 0.80; monthly average: 0.83; quarterly average: 0.82), followed by the Ordinary Kriging algorithm (0.79, 0.82, and 0.82, respectively), and finally the Inverse Distance Weighted algorithm (0.78, 0.81, and 0.80, respectively).

**Table 2:** Comparison of interpolated results derived from Information Diffusion, Inverse Distance Weighted, Ordinary Kriging respectively.

Daily Average	Information Diffusion				Inverse Distance Weighted				Ordinary Kriging			
	R^2^	MAE (µg/m^3^)	RMSE (µg/m^3^)	RAE (µg/m^3^)	R^2^	MAE (µg/m^3^)	RMSE (µg/m^3^)	RAE (µg/m^3^)	R^2^	MAE (µg/m^3^)	RMSE (µg/m^3^)	RAE (µg/m^3^)
Mean	0.80	13.81	19.05	68.05	0.78	14.40	20.22	74.31	0.79	13.56	18.90	67.71
Var	—	1.05	2.55	153.78	—	1.99	4.66	526.54	—	1.05	2.33	265.66
**Monthly Average**												
Mean	0.83	11.01	15.64	57.80	0.81	11.50	16.69	63.52	0.82	11.55	16.18	58.74
Var	—	1.15	2.84	247.79	—	1.31	2.64	527.98	—	2.03	5.59	370.70
**Quarterly Average**												
Mean	0.82	8.78	11.97	39.61	0.80	9.21	12.70	44.03	0.82	8.79	11.95	39.56
Var	—	0.82	1.31	16.82	—	1.72	4.14	89.17	—	1.37	2.76	69.43

Estimates using the information diffusion algorithm showed $\overline{RMSE}$ (mean value of RMSE) values that were 0.73 µg/m^3^ to 1.17 µg/m^3^ smaller than those of the Inverse Distance Weighted algorithm (Table 2). The $\overline{RMSE}$ values of daily average and quarterly average results of information diffusion algorithm were larger than those of Ordinary Kriging, but the differences were relatively small. According to the $\overline{MAE}$ and $\overline{RAE}$ analysis results, all results except daily average data showed that information diffusion algorithm results were more reliable. Specifically, information diffusion results based on monthly average data showed higher accuracy.

Due to the impacts of algorithm principles, the estimation results of the three algorithms varied in some details. In general, using the information diffusion algorithm, estimated values beyond a PM_2.5_ concentration interval of 35–115 µg/m^3^ were smaller than monitoring values, while estimates within the interval of 35–115 µg/m^3^ were larger than monitoring values (Table 3). Moreover, the distribution of estimation results in each interval varied with time scale for the other two algorithms (Table 3). Estimation errors of the information diffusion algorithm typically fluctuated within a smaller range, showing higher stability than the other two methods (Tables 2 and 3).

**Table 3:** Variations in different grades of PM_2.5_ concentrations estimated by different methods.

**Daily Average** (**µg/m** ^**3**^)	0–35	35–75	75–115	115–150	150–250	250–350
Ground-measured PM_2.5_ concentration	10.00%	47.00%	29.00%	4.00%	9.00%	1.00%
Information Diffusion	9.00%	44.00%	34.00%	5.00%	8.00%	0.00%
Inverse Distance Weighted	10.00%	42.00%	34.00%	7.00%	6.00%	1.00%
Ordinary Kriging	6.00%	45.00%	35.00%	8.00%	5.00%	1.00%
**Monthly Average** (**µg/m** ^**3**^)						
Ground-measured PM_2.5_ concentration	13.00%	36.00%	42.00%	6.00%	3.00%	0.00%
Information Diffusion	14.00%	28.00%	52.00%	4.00%	2.00%	0.00%
Inverse Distance Weighted	15.00%	34.00%	42.00%	6.00%	3.00%	0.00%
Ordinary Kriging	14.00%	30.00%	47.00%	6.00%	3.00%	0.00%
**Quarterly Average** (**µg/m** ^**3**^)						
Ground-measured PM_2.5_ concentration	6.00%	36.00%	50.00%	8.00%	0.00%	0.00%
Information Diffusion	1.00%	37.00%	56.00%	6.00%	0.00%	0.00%
Inverse Distance Weighted	2.00%	32.00%	61.00%	5.00%	0.00%	0.00%
Ordinary Kriging	3.00%	40.00%	50.00%	7.00%	0.00%	0.00%

#### Analysis of PM_2.5_ spatial distribution

**Figure 4:**
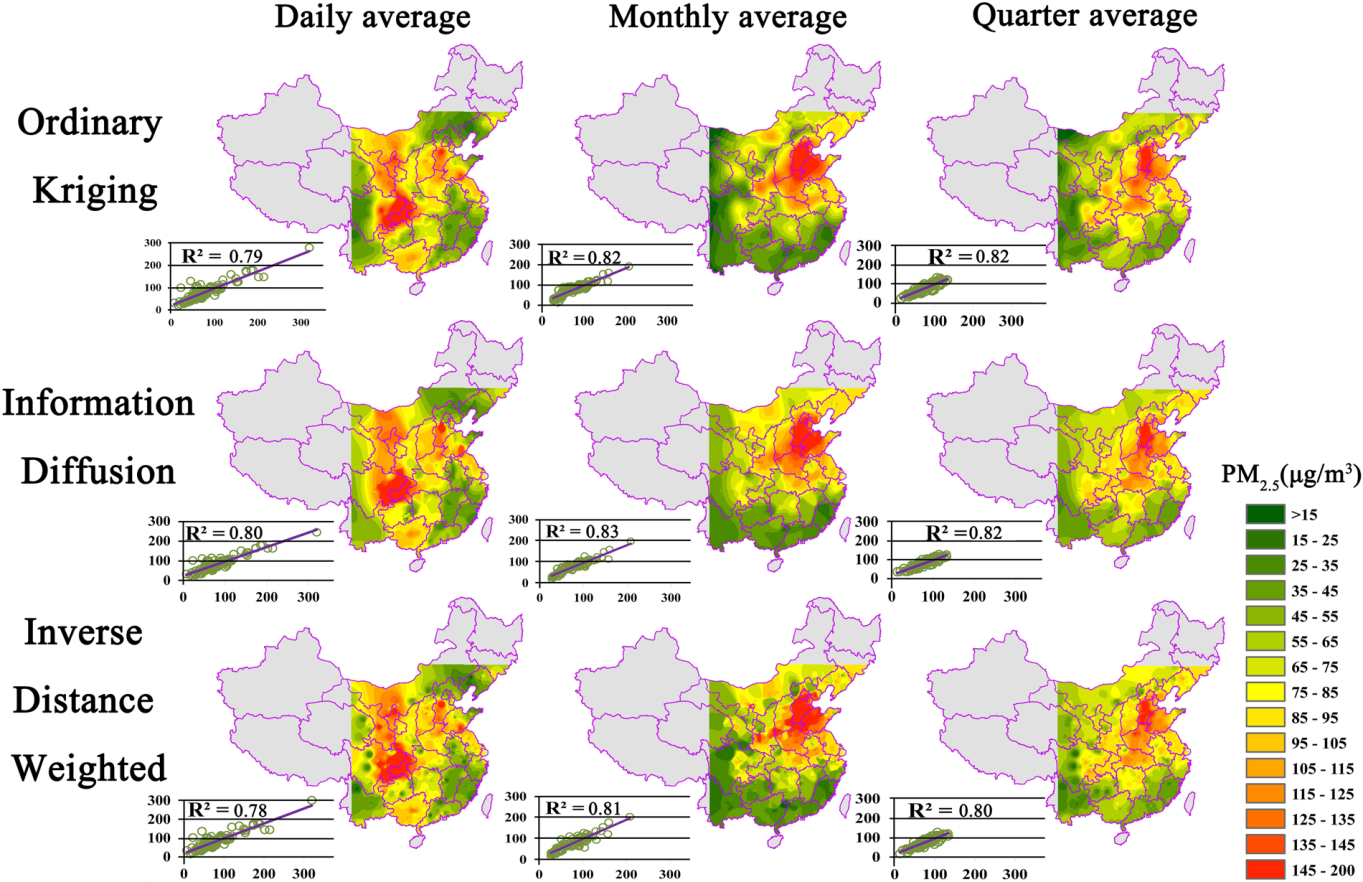
Spatial distribution of PM_2.5_ concentration in the study area using Ordinary Kriging, Information Diffusion, and Inverse Distance Weighted algorithms for different time scales. (Created by ArcMap, version 10.2, http://www.esri.com/).

Ground PM_2.5_ concentrations estimated by the above three algorithms are illustrated in Fig. 4. The daily, monthly, and quarterly average PM_2.5_ concentrations obtained by different algorithms showed similar spatial distributions. In the daily average results, PM_2.5_ concentrations were higher than 145 µg/m^3^ in central Hebei, southeast Shandong, north Ningxia, Chongqing, and east Sichuan. In the monthly average results, PM_2.5_ concentrations were high in central and south Hebei, west Shandong, north Henan, south Shaanxi, and central Liaoning. The spatial distribution of high PM_2.5_ concentrations using the quarterly average results was similar to that using the monthly average results. In the estimation results for all time scales, high PM_2.5_ concentrations were similarly spatially distributed, occurring in northeastern and eastern coastal areas of China. In addition, PM_2.5_ concentrations were high in densely populated and urban built-up regions such as Sichuan Basin and Guanzhong Plain, and were relatively low in high-altitude regions such as the Tibetan Plateau and the Yunnan-Guizhou Plateau. PM_2.5_ concentrations were also relatively low in southeastern coastal areas with abundant average annual precipitation, despite this including the densely populated and built-up urban region of the Pearl River Delta.

Whilst similar, the spatial distribution results of the three algorithms showed certain differences. Due to the impacts of algorithm principles, the results obtained by the Inverse Distance Weighted algorithm indicated laminar diffusion from the monitoring sites in the center to their surroundings. The estimation results of the Ordinary Kriging algorithm showed this phenomenon to a lesser extent; however, this algorithm considered that only one factor affected PM_2.5_ spatial distribution, i.e. the relationships between monitoring data, thus leading to a concentric diffusion of estimation results. The information diffusion algorithm corrected such phenomena to some extent; therefore, variations in estimation results were smoother than the Inverse Distance Weighted algorithm, and did not show any clear concentric diffusion. In addition, the extreme values in the estimation results of the information diffusion algorithm were less than those of the other two algorithms, indicating that our proposed method had a smoothing effect on extreme values.

#### Features of the DEM-assisted information diffusion algorithm

The results showed that PM_2.5_ concentrations in high-altitude regions (Tibetan Plateau, Yunnan-Guizhou Plateau, north Sichuan, northwest Hubei, and southwest Hubei) were lower than those of surrounding areas (Fig. 4), indicating a clear negative correlation between PM_2.5_ concentration and elevation.

**Figure 5:**
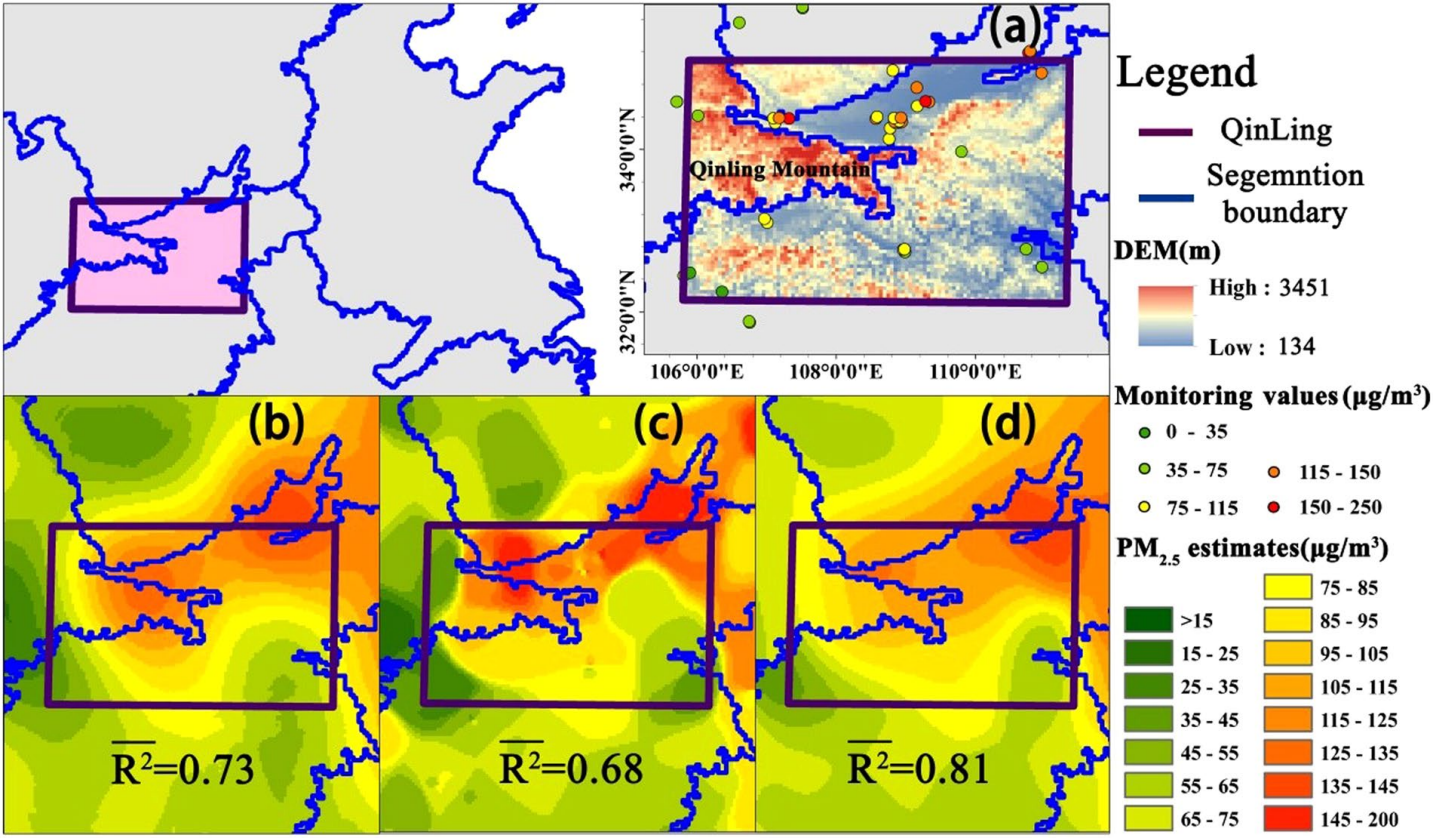
Map showing (**a**) the terrain of Qinling, and (**b**–**d**) the spatial distribution and correlations of monthly average PM_2.5_ concentration in Qinling produced by (**b**) Ordinary Kriging, (**c**) Inverse Distance Weighted, and (**d**) Information Diffusion algorithms. (Created by ArcMap, version 10.2, http://www.esri.com/).

Using the information diffusion algorithm, this study considered the impact of elevation on PM_2.5_ spatial distribution, and segmented the study area based on elevation variations. Based on estimation results, this algorithm revealed a greater impact of elevation than the other two algorithms. We performed a comparative analysis on south Shaanxi and peripheral areas of the Qinling Mountains, shown in Fig. 5 as the rectangular area called Qinling area. The highest altitude in this area exceeds 3400 m (the red area in Fig. 5a, called Qinling Mountain). The northern area is Guanzhong Plain (Guanzhong urban agglomeration, where cities and people are clustered) and the southern area is Ankang Basin, which have different altitudes. The blue line is the segmentation boundary. Monitoring values in Qinling Mountain were relatively low, and were higher in Guanzhong Plain. Estimates based on the three methods showed similar patterns generally in global area (Fig. 4), but spatial distributions of ground PM_2.5_ concentrations varied greatly in local area of complex terrain (Fig. 5). Estimates were relatively high in Guanzhong Plain but in high-altitude regions (Qinling Mountain), the information diffusion algorithm estimated significantly lower PM_2.5_ estimates, while other methods that did not consider the effect of terrain still estimated relatively high PM_2.5_ concentrations. Qinling Mountains play an important role in preventing PM_2.5_ diffusion, resulting in no high PM_2.5_ concentration areas. This suggests that the information diffusion algorithm estimates based on DEM segmentation more accurately reflect the real spatial distribution of PM_2.5_ than traditional interpolation algorithms.

## Discussion

By combining the advantages of DEM data and the information diffusion algorithm, this study developed a DEM-assisted information diffusion algorithm, which we have proved is a powerful tool for estimating ground PM_2.5_ concentrations. The DEM-assisted information diffusion algorithm reduced the algorithm dependence on the distribution characteristics of monitoring sites, and improved the performance of the estimation algorithm. According to our results, estimates obtained by the information diffusion algorithm were more accurate than those obtained by traditional interpolation algorithms.

### Comparison between the information diffusion algorithm and traditional algorithms

Although the ground PM_2.5_ monitoring network has become increasingly sophisticated, the inhomogeneous spatial distribution of monitoring sites and lack of data affect the estimation accuracy of traditional interpolation algorithms^23^. As traditional interpolation algorithms only consider the relationships between sample data, the estimates obtained by such algorithms have obvious defects and usually display concentric spatial distributions, which do not comply with PM_2.5_ diffusion rules^26^. This phenomenon shows that traditional algorithms are dependent on the distribution (site density and mutual distance) of monitoring sites^15^. Thus, the information diffusion algorithm is more reliable when processing small-sample data^24^. In addition, DEM multi-scale segmentation divides the study area into different segments, and a buffer is built for each segment, reducing abnormal phenomena such as abrupt variations and defects in PM_2.5_ concentrations. Thus, the method proposed in this study reduces the dependence of the algorithm on the distribution characteristics of monitoring sites. Subsequently, an accurate estimation and mapping of PM2.5 concentrations could benefit more from the proposed method, which is able to delineate the distribution pattern of PM2.5 concentrations finely by high resolution interpolation at local region (e.g., 1 km resolution in this study).

The information diffusion algorithm was slightly superior to traditional interpolation algorithms based on the accuracy assessment of whole monitoring sites (Table 2 and Fig. 4). The information diffusion algorithm error was relatively small, the correlation was 0.01–0.02 higher than other algorithms slightly, and the R^2^ of the estimated monthly average ground PM_2.5_ concentrations reached 0.83. Furthermore, the proposed method considering the DEM has a big advantage at local region of complex terrain, while the R^2^ for the proposed method is significantly better than that of other both methods, respectively 0.81 for the proposed method, 0.73 for Ordinary Kriging method and 0.68 for inverse Distance Weighted method (Fig. 5). This is mainly attributed the subareas divided by segmenting the DEM that they pay more attention to the monitoring points within themselves, while it has been proved that PM2.5 distribution is related to landscape and is region-dependence^41^. Thus, PM_2.5_ concentrations estimated by the DEM-assisted information diffusion algorithm are highly reliable.

Previous studies have suggested that ground PM_2.5_ concentrations are linked to multiple factors, and the impact factor varies with location^22^. However, in most studies, ground PM_2.5_ concentrations are highly correlated to elevation^22,42^, while elevation is evidently collinear with economic development level^1^. This study used DEM segmentation results to represent the economic development level of each segmented region, and regarded the edges of these regions as hard break lines during calculation, overcoming the difficulty in spatial quantification of economic and social data. Compared with traditional interpolation algorithms, the DEM-assisted information diffusion algorithm proposed in this study only requires commonly available DEM data, to obtain the high resolution interpolation PM2.5 concentrations, especially for local area. This algorithm can obtain more accurate estimation results without acquiring optimal parameter values or transforming the normal distribution of data, making it more applicable than the other two algorithms compared in this study.

### Analysis of estimation results based on different time scales

The estimation accuracy obtained by three methods differed significantly with time scale. The accuracy of estimates using quarterly average data was better than that of estimates using daily and monthly average data (Table 2). It could be attributed to the data quality improvement due to the average of long time series monitoring data. On the other hand, it seemed that information diffusion algorithm perform better for daily average data, even though the accuracies of three methods using daily data are all low. The main reason for this might be the smoothing effect of the information diffusion algorithm on extreme values, while the variation of RAE for the information diffusion method is superior obviously to other both methods (Table 2). Furthermore, monthly average data not only reduced the influence of extreme values on estimation results, but also kept the data from being over-average. With extending the time scale, the advantages of the proposed method become unapparent, especially for quarterly average data. Thus, this study suggests that the information diffusion algorithm is more applicable for estimating daily/monthly average monitoring data. Subsequently, our method could contribute more to the mapping of high temporal resolution monitoring data.

### Analysis of PM_2.5_ spatial distribution patterns

PM_2.5_ concentrations showed significant spatial heterogeneity (Fig. 4), which might be related to emission factors and dispersal conditions^41^. In winter, regions with high ground PM_2.5_ concentrations are the northeastern area, eastern coastal area, and Guanzhong urban aggolmeration^22^. In the densely populated and built-up urban area of Sichuan Basin, the ground PM_2.5_ concentration is much higher than in peripheral areas. Low precipitation, heavy pollution due to coal burning, developed industries, high urbanization, and excessive use of motor vehicles could explain the poor air conditions^4,43,44^. In the middle of Inner Mongolia and Shanxi province, despite relatively high altitudes, dust originating from the Loess plateau and the mixed barren soil often degrades air quality^17,45^. Coal burning could also be a dominant cause of air pollution in these areas, especially during winter. Additionally, dispersal conditions related to the topography could affect the distribution of cold and hot points^1^. Meanwhile, in the southeastern coastal area with abundant precipitation, and high-altitude regions such as the Tibetan Plateau and the Yunnan-Guizhou Plateau, ground PM_2.5_ concentrations are low.

In summary, due to the imperfect ground PM_2.5_ monitoring network, common ground PM_2.5_ concentration inversion models require significant supplementary data to obtain higher inversion accuracy (for example, terrain, weather, and economic development data)^4,13^. The high spatial heterogeneity of supplementary data makes it very difficult to apply and develop these models^13^. The information diffusion algorithm only requires DEM data; therefore, its maturity and wide application reduces difficulties relating to algorithm development. Nevertheless, we still expect that the new models would be developed to integrate all relevant factors (e.g., land use, landscape pattern, population, meteorological variables), while it is already proved further that PM_2.5_ concentration is related to land use and the corresponding landscape, especially for high resolution mapping at regional scale^44,46^.

## Conclusions

To address the common spatial heterogeneity issue of monitoring data used in atmospheric science studies, this research applied the information diffusion algorithm to ground PM_2.5_ concentration estimation. The results showed that, compared with traditional interpolation algorithms, the DEM-assisted information diffusion algorithm focuses on the impact of elevation factors on PM_2.5_ spatial distribution, and is not restricted by the lack of monitoring sites and their inhomogeneous spatial distribution. Therefore, the estimation results of this algorithm are more accurate and more appropriately distributed than those of traditional interpolation algorithms, to contribute to the high temporal resolution mapping. Using DEM segmentation results as supplementary information, estimates using this algorithm are more compliant with the diffusion rules of air pollutants. However, the proposed method only considered the effect of topographic characteristic. In the future research, we expect that an advanced method is developed to consider more factors when conducting the interpretation based on the station-based PM_2.5_ monitoring data, since the spatial distribution of PM2.5 concentrations is strongly impacted by many factors, for example, landscape type, economic development level, and so on.
